# Supplementary material for: Using Cryo-ET to distinguish platelets during pre-acute myeloid leukemia from steady state hematopoiesis
Source: Commun Biol. 2022 Jan 20;5:72. doi: 10.1038/s42003-022-03009-4 (PMC8776871; doi:10.1038/s42003-022-03009-4)
Supplement: Supplementary file 3 — Description of Additional Supplementary Files [file 42003_2022_3009_MOESM3_ESM.pdf]

Description of Additional Supplementary Files

File name: Supplementary Movie 1

Description: Representative healthy platelet tomogram. Slicewise view scrolling through the tomogram. Organelles are annotated.

File name: Supplementary Movie 2

Description: Representative AML platelet tomogram. Slicewise view scrolling through the tomogram. Organelles are annotated.

File name: Supplementary Movie 3

Description: Representative pre-AML platelet tomogram. Slicewise view scrolling through the tomogram. Organelles are annotated.

File name: Supplementary Data 1

Description: Average platelet and mitochondria areas in un-irradiated WT, Pre-AML, AML, and controls.

File name: Supplementary Data 2

Description: Circularity measurements of abnormal and normal mitochondria.

File name: Supplementary Data 3

Description: Source data for Fig. 3d.
