# Supplementary material for: Using Cryo-ET to distinguish platelets during pre-acute myeloid leukemia from steady state hematopoiesis
Source: Commun Biol. 2022 Jan 20;5:72. doi: 10.1038/s42003-022-03009-4 (PMC8776871; doi:10.1038/s42003-022-03009-4)
Supplement: Supplementary file 10 — Reporting Summary [file 42003_2022_3009_MOESM10_ESM.pdf]

nature portfolio

Corresponding author(s): Zhao Wang

Last updated by author(s): Oct 25, 2021

Reporting Summary

Nature Portfolio wishes to improve the reproducibility of the work that we publish. This form provides structure for consistency and transparency in reporting. For further information on Nature Portfolio policies, see our Editorial Policies and the Editorial Policy Checklist.

Statistics

For all statistical analyses, confirm that the following items are present in the figure legend, table legend, main text, or Methods section.

n/a	Confirmed	
☐	☒	The exact sample size (*n*) for each experimental group/condition, given as a discrete number and unit of measurement
☐	☒	A statement on whether measurements were taken from distinct samples or whether the same sample was measured repeatedly
☐	☒	The statistical test(s) used AND whether they are one- or two-sided<br>*Only common tests should be described solely by name; describe more complex techniques in the Methods section.*
☐	☒	A description of all covariates tested
☐	☒	A description of any assumptions or corrections, such as tests of normality and adjustment for multiple comparisons
☐	☒	A full description of the statistical parameters including central tendency (e.g. means) or other basic estimates (e.g. regression coefficient) AND variation (e.g. standard deviation) or associated estimates of uncertainty (e.g. confidence intervals)
☐	☒	For null hypothesis testing, the test statistic (e.g. *F*, *t*, *r*) with confidence intervals, effect sizes, degrees of freedom and *P* value noted<br>*Give P values as exact values whenever suitable.*
☒	☐	For Bayesian analysis, information on the choice of priors and Markov chain Monte Carlo settings
☒	☐	For hierarchical and complex designs, identification of the appropriate level for tests and full reporting of outcomes
☒	☐	Estimates of effect sizes (e.g. Cohen's *d*, Pearson's *r*), indicating how they were calculated

Our web collection on statistics for biologists contains articles on many of the points above.

Software and code

Policy information about availability of computer code

Data collection	SerialEM, Illumina Sequencer
Data analysis	EMAN2, UCSF Chimera, UCSF ChimeraX, STAR (version 2.5.2b), DESeq2 (version 1.12.4), SSPS 16.0

For manuscripts utilizing custom algorithms or software that are central to the research but not yet described in published literature, software must be made available to editors and reviewers. We strongly encourage code deposition in a community repository (e.g. GitHub). See the Nature Portfolio guidelines for submitting code & software for further information.

Data

Policy information about availability of data

All manuscripts must include a data availability statement. This statement should provide the following information, where applicable:

- Accession codes, unique identifiers, or web links for publicly available datasets
- A description of any restrictions on data availability
- For clinical datasets or third party data, please ensure that the statement adheres to our policy

EMD-24287, EMD-24291, EMD-24298, EMD-24289 and EMD-24288

Field-specific reporting

Please select the one below that is the best fit for your research. If you are not sure, read the appropriate sections before making your selection.

☒ Life sciences ☐ Behavioural & social sciences ☐ Ecological, evolutionary & environmental sciences

For a reference copy of the document with all sections, see nature.com/documents/nr-reporting-summary-flat.pdf

Life sciences study design

All studies must disclose on these points even when the disclosure is negative.

Sample size	We did not pre-determine the sample size. We found that abnormal mitochondria were only present in pre-AML platelets but not un-irradiated or control platelet. It is rather an observation than a statistical difference. In addition, based on a power analysis, we believed our sample size was sufficient to draw the current conclusion.
Data exclusions	Platelets in sub-optimal ice or on the edge of the mesh were excluded from data collection.
Replication	We collected data for 3 mice in a group in parallel, which was a replication for each experiment.
Randomization	The mice were picked randomly to conduct this experiment. Blood drawn, EM specimen preparation and platelet data collection were random procedures to rule out the possibility of any bias and preference.
Blinding	Different groups of platelets were first visually confirmed locations on the grids by human. This step only provide location for tomogram collectiion with no ultra-structual feature could be seen until reconstruction was done.

Reporting for specific materials, systems and methods

We require information from authors about some types of materials, experimental systems and methods used in many studies. Here, indicate whether each material, system or method listed is relevant to your study. If you are not sure if a list item applies to your research, read the appropriate section before selecting a response.

Materials & experimental systems

n/a	Involved in the study	
☒	☐	Antibodies
☒	☐	Eukaryotic cell lines
☒	☐	Palaeontology and archaeology
☐	☒	Animals and other organisms
☒	☐	Human research participants
☒	☐	Clinical data
☒	☐	Dual use research of concern

Methods

n/a	Involved in the study	
☒	☐	ChIP-seq
☒	☐	Flow cytometry
☒	☐	MRI-based neuroimaging

Animals and other organisms

Policy information about studies involving animals; ARRIVE guidelines recommended for reporting animal research

Laboratory animals	CD45.1 or or C57BL/6 mice, Female
Wild animals	None
Field-collected samples	None
Ethics oversight	Animal care facilities at Baylor College of Medicine (BCM). All procedures were approved by the BCM Institutional Animal Care and Use Committee.

Note that full information on the approval of the study protocol must also be provided in the manuscript.
